# An Emulation Model Combining Caries and Periodontitis Risk Assessment With Student Performance Report: An Exploratory Project

**DOI:** 10.1002/cre2.70135

**Published:** 2025-04-30

**Authors:** David Johnsen, Aditi Jain, Carlos Garaicoa‐Pazmino, Justine Kolker, Megumi Williamson, Wei Shi

**Affiliations:** ^1^ Department of Pediatric Dentistry University of Iowa College of Dentistry and Dental Clinics Iowa City Iowa USA; ^2^ Department of Operative Dentistry University of Iowa College of Dentistry and Dental Clinics Iowa City Iowa USA; ^3^ Department of Periodontics University of Iowa College of Dentistry and Dental Clinics Iowa City Iowa USA; ^4^ School of Dentistry Universidad de Especialidades Espiritu Santo Samborondon Ecuador; ^5^ Department of Periodontics East Carolina University School of Dental Medicine Greenville North Carolina USA; ^6^ Iowa Oral Health Research Institute University of Iowa Iowa City Iowa USA

**Keywords:** critical thinking, dental caries, periodontitis, risk assessment

## Abstract

**Objectives:**

Patient risk assessments for caries and periodontitis are hourly activities for dentists. The present study tested a caries and periodontitis risk assessment format based on managing the person using a critical thinking emulation model including levels of patient capacity and disease management. Additionally, we explored factors in student analysis contributing to the designation of higher/lower‐risk determinants for caries and periodontitis.

**Material and Methods:**

Third‐year dental students assessed patient risk for recurring disease in periodontics and cariology exercises. The risk assessment thought process becomes the learning outcome, learning guide, and assessment instrument. Students demonstrated the risk assessment analysis with a patient using PowerPoint presentations.

**Results:**

Students applied over 95% of procedural steps (e.g., exam, history) with a range of judgmental steps (65%–100%) in risk level, interprofessional practice, disease progression, compliance, among others. Designation of higher and lower‐risk determinants for periodontitis risk assessment (*n* = 38), students identified differences in patient risk based on whether the patient had diabetes or smoked (*p* = 0.013 to < 0.001). For caries risk assessment (*n* = 40), students identified differences between lower‐ and higher‐risk patients for patient compliance (*p* = 0.001), behavior control (*p* = 0.006), diet control (*p* < 0.001), and prognosis (*p* = 0.005).

**Conclusions:**

The combined learning guide warrants further exploration in guiding dental students' risk analysis for periodontitis and caries by emulating the thinking process of a master clinician. Future work includes probing analysis with binary conclusions, interventions for high‐risk people with recurring disease, and the association of treatment and health.

## 1 Introduction

**Figure 1:**
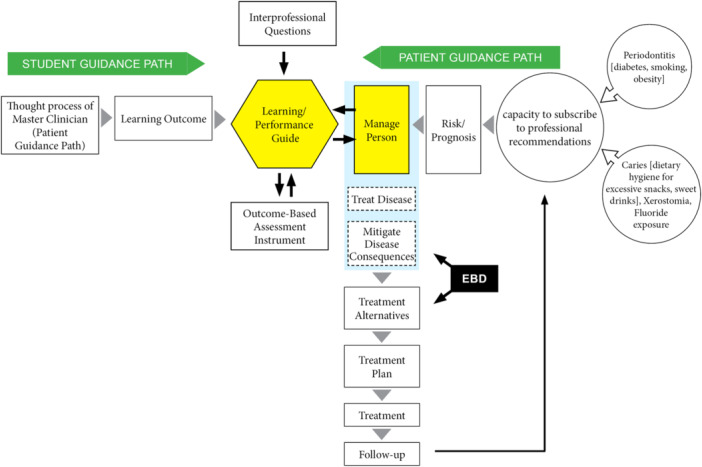
Concept map for a combined caries/periodontitis risk assessment. The concept map has a Patient Guidance Path starting on the right side, converging with a Student Learning Path starting on the left side and converging at the points of “Learning/Performance Guide” and “Manage the Person.” The Patient Guidance Path begins with a risk assessment for periodontitis, starting with major risk factors to include diabetes, smoking, and obesity, and a risk assessment for caries, starting with excessive sweets or xerostomia. Risk assessment proceeds through the patient's capacity to subscribe to professional recommendations to a risk/prognosis, leading to risk assessment based on managing the person. The Student Guidance Path begins with deriving the thought process of the master clinician succinctly enough for the student to apply to the next person. The thought process of the master clinician becomes the learning outcome, the learning/performance guide, and the assessment instrument. Noted is the connection (dotted enclosure) among managing the person, treating the disease, and mitigating the symptoms.

An essential hourly activity for dentists is to assess patient risk for caries and periodontitis as a basis for treatment planning and for long‐term follow‐up to maximize sustained patient health (Shabayek et al. 2023). While the pathogenesis and treatment of dental caries and periodontitis are distinctly different, the thought processes in conducting risk assessment are essentially identical (Figure 1). The overarching idea in Figure 1 is to articulate the main points for the thought process in risk assessment and treatment planning. This streamlining of the thinking model can then become the learning outcome, a learning guide, and an assessment instrument.

A first step in the risk assessment thought process, for either caries or periodontitis, is for the practitioner to identify major risk factors; the thought process for identifying major risk factors is essentially identical once baseline data are gathered. For periodontitis, major risk factors include diabetes mellitus, smoking, obesity, poor oral hygiene, among others (Lang and Tonetti 2003). For dental caries, major risk factors include unrestricted cariogenic diet and dietary hygiene, xerostomia, lack of fluoride exposure, etc. (Nunes et al. 2014; Weyant et al. 2013). Once major risk factors are identified, the risk assessment thought processes for caries and periodontitis become indistinguishable.

The second step is to determine the capacity of the patient to subscribe to professional recommendations (T. W. Craig et al. 2022; Shabayek et al. 2023). Success in this assessment is a basis for accurately projecting risk and prognosis. A limited patient capacity can immediately place the patient at higher risk.

A third step is meant to assign risk and prognosis. One viable approach is to combine immediate risk with disease progression (T. Craig et al. 2020). This approach can change the paradigm from a snapshot to a moving picture. The lower‐risk person is likely to have minimal disease. An individual with a higher risk may have minimal disease associated with a medical/life event (e.g., myocardial infarction, dementia, Parkinson's disease, or a sudden lifestyle change). Ultimately, the higher‐risk person with co‐destructive risk factors (e.g., diabetes mellitus, smoking, cariogenic diet) can have an exacerbated risk with a chronically progressing disease. The prognosis will depend on the severity of the risk factors, the ability of the practitioner to control them, and patient's compliance to treatment recommendations.

The final step is to confront the disease (caries or periodontitis) at three different levels: manage the person, treat the disease, and mitigate the disease consequences. Mitigating the disease consequences can include caries removal and restoration of tooth substance and/or providing periodontal therapy (e.g., scaling and root planing with or without periodontal surgery). Treating the disease includes increased exposure to fluoride for caries recurrence or patient education on optimal oral hygiene practices. Managing the person means dealing with social determinants and barriers to care (T. Craig et al. 2020; T. W. Craig et al. 2022).

A question arises whether mitigating the disease consequences without managing the person can bring a sustained health. This risk assessment model leads the student to try to manage the person before attempting to treat the disease or mitigate disease consequences, followed by a proposal of reasonable treatment plan and alternatives supported by principles of evidence‐based dentistry (EBD) at each of the steps. As such, the outline of the proposed learning guide is a detail of the risk assessment thought process in Figure 1. Concepts in critical thinking applied to risk assessment emulates the intended activity (what the student is to do and how the student thinks when taking the risk assessment thought process to the patient), gain agreement of faculty on content, application, and assessment of the thought process to the patient and use the same instrument to guide learning and assess performance (Johnsen et al. 2012; Marshall et al. 2017).

The primary objective of the present project is to apply concepts from an emulation model in critical thinking to a combined risk assessment for caries and periodontitis, with a report on student performance. With both higher‐ and lower‐risk patients in student exercises, a secondary purpose is to explore factors in the line of student analysis contributing to the designation of higher risk and lower risk for caries and periodontitis using this model.

## 2 Materials and Methods

The present study was granted an “Exempt status” by the University of Iowa Institutional Review Board (IRB #202208293).

### 2.1 Academic Setting

Paralleling previous work in Geriatrics (T. Craig et al. 2020; Marchini et al. 2017), third year (D3) dental students in Periodontics and Operative Dentistry were offered a PowerPoint template parallel to the learning guide. A student profile for third year (D3) student means the student has had patient care starting in D1 with preventive care, mostly prophylaxis and scaling, with follow‐ up patient care in D2 in minor restorative dentistry. In D3, students have clerkship rotations in Prosthodontics, Endodontics, Pediatric Dentistry, and Oral Surgery, in addition to Periodontics and Operative Dentistry/Cariology. After a comprehensive medical and dental exam, D3 students entered patient information and responses to questions on the template and present their patients in a seminar. Sections of the Learning Guide are depicted in (Appendix A) including multiple sections listed as “Patient History” with four steps, “Patient Examination” with four steps, “Risk Assessment” with seven steps, and “Treatment” with four steps. Evidence‐based dentistry (EBD) has four steps, summarized to one step for this presentation. Single steps in the Learning Guide are “Prioritize Conditions,” “Prognosis Without Treatment,” “Evaluation of Outcomes,” and “Self‐Assessment.” The Learning Guide and the PowerPoint template have identical headings with the intent of being complimentary, consistent, and reinforcing. The student makes entries on each heading of the PowerPoint to report on the application of the content of the heading relative to the student's patient. The faculty makes entries on each heading on the learning guide/assessment instrument relative to student performance. The faculty circles the “A” if the student applied the step and circles the “G” if the student grasps the concept of the step.

Data were obtained from learning guides among 78 dental students (38 in Periodontics [periodontitis] and 40 in Operative Dentistry [dental caries]) serving as the basis for the present study. Most PowerPoint presentations in Operative Dentistry and Periodontics had more than 35 slides with extensive details. For example, all contributory and noncontributory medical conditions, environmental habits, and medications are listed with interactions and/or impact within the oral cavity. Risk assessment is presented in the context of a more comprehensive learning guide to include data gathering, EBD, interprofessional practice (IPP) questions, treatment follow‐up, and biases. Caries risk assessment has over 15 questions to consider.

Students in each discipline were divided into 4 subgroups that consisted of up to 10 students per subgroup. In addition to hearing the analysis of their patient, each student witnesses the analyses of nine other students.

### 2.2 Assessment of Critical Thinking Skills

The learning guide/assessment instrument (Appendix A) is abbreviated from the data inserted by the students on the PowerPoint presentations. Critical thinking skill is based on mastery of the thought process depicted in Figure 1 graphically and articulated in more detail in the learning guide. Emulation of a designated thought process develops alternatives and is different from a decision tree, where there is an “answer”; this emulation leads to alternatives.

This project combined caries and periodontitis risk. Concepts in critical thinking in other areas of patient analysis were adapted to a combined analysis for caries and periodontitis risk (Marchini et al. 2017; Marshall et al. 2017). For validity, we are not aware of a previous project combining a person‐based risk assessment for caries and periodontitis using an emulation model for critical thinking. Students conducted a probing analysis with extensive data funneling to binary conclusions, calling on thinking and judgment for risk level, compliance, diet, and systemic conditions. As students systematically ask insightful questions in accord with the learning guide, they will get better at developing sound alternatives with experience. Feasibility of the current project is based on separate implementations of previous projects using an emulation model (T. Craig et al. 2020; T. W. Craig et al. 2022).

While students spend extended time on medical/dental histories and examinations for their presentations, data for the development of this learning program were gathered from students from their examinations or from patient interviews. All data were verified by faculty in the clinic, and all judgments offered by students in the seminars were scrutinized by faculty during direct patient care. Thus, the accuracy of the data depends on the student or the student gaining reliable information from the patient. The most reliable information is expected from oral exams and histories, where numbers are available. Entries on compliance, risk level, prognosis, and diet reflect the student's judgment with faculty verification in the clinic and faculty scrutiny in the seminar. The most vulnerable data are likely from patient recall of time frames, such as number of years of smoking. For this project, the emphasis is on risk assessment. While data are available on other areas (e.g., demographics, scrutiny of technical procedures, IPP, EBD, results, and discussion for this project focused on risk assessment and implications, not on EBD and IPP.

Students completed the analysis, followed by questions of fellow students and faculty. Students who complete all entries in the template are almost all judged to demonstrate mastery of the basic skillset/outcome for respective stage of development.

### 2.3 Data Management Issues Resulting From Combination of Risk Assessments for Caries and Periodontitis

In both Periodontics and Operative Dentistry sessions, the patient pools include higher‐ and lower‐risk patients. Thus, students were required to differentiate between higher‐ and lower‐risk patients and directed to select a patient needing comprehensive care from their respective clerkships. Patients were not screened by faculty before the presentations. The mix of patients in both sets of presentations is not representative of the general population. In Periodontics, the two main prototypes were patients with periodontitis in need of active periodontal therapy and healthy patients seeking routine periodontal procedures (e.g., implant therapy, crown lengthening, soft tissue augmentation). For Operative Dentistry, the two main prototypes were patients with high caries rates and patients seeking some routine restorative procedures (e.g., restorations in the esthetic zone, bleaching). Low‐risk patients tended to have a favorable prognosis and are well‐managed, whereas higher‐risk patients had a less favorable prognosis and thus, requiring mitigation just as much as disease management.

In exploring key variables for higher‐risk patients in Periodontics, student entries were analyzed for risk level, disease onset, compliance capacity, and prognosis for patients with diabetes, smoking history, or obesity versus patients with no diabetes, smoking history, or obesity. In exploring key variables for caries, student entries were analyzed for risk level, current caries experience, diet, compliance capacity with accompanying medical, socioeconomic, or behavioral conditions.

Seminars are preceded by mastery of extensive didactic material and simulated procedural proficiency. Didactic instruction includes healthy and diseased states for dental caries and periodontitis, concepts in managing localized disease, specific risk factors for caries and periodontitis, systemic conditions, socioeconomic status, and behavioral contributors for health and disease.

Student entries presented in the seminar, rather than commentary by faculty, are the sources of data. Thus, the use of data is for the development of learning tools and not to add to the science of disease understanding. While the learning guides are almost identical for both specialties, data are reported separately for Operative Dentistry and Periodontics. For example, students in Operative Dentistry performed a comprehensive periodontal exam, including parameters such as probing depths, bleeding on probing, etc. Similarly, students evaluated caries risk assessment during presentations in Periodontics.

Disease progression designation follows that used in Geriatrics: risk level 1 – “Minimal risk, Minimal disease progression,” risk level 2 – “High risk, minimal disease progression,” risk level 3 – “High risk, progressing disease,” and risk level 4 – “Destruction has happened” (T. Craig et al. 2020; Marchini et al. 2017).

### 2.4 Statistical Analysis

The statistical analyses were focused on exploring factors in student analysis contributing to the designation of higher risk and lower risk for caries and periodontitis using this emulation model. Univariate logistic regression analysis was performed to assess the association between patients' health status (e.g., smoking, diabetes, obesity) and health providers' risk assessment (e.g., compliance, disease, risk and prognosis) among dental caries and periodontitis patients. Wald Chi‐square test was used to test the significance of association. The Chi‐square test was used to show the likelihood of results being due to a random distribution. *p*‐values of ≤ 0.05 were considered statistically significant, and differences were noted by asterisks (*) (*p ≤ 0.05, **p ≤ 0.01, ***p ≤ 0.001, and *p* > 0.05, not significant).

## 3 Results

### 3.1 Overall Performance for Procedural and Judgment Aspects for Patient Assessment

While the primary focus was on risk assessment for caries and periodontitis using an emulation model, student performance was also recorded for remaining steps in the learning guide (Table 1). Students had higher application overall for the more procedural steps of “Patient History” and “Patient Examination” than for steps involving more thinking and judgment in risk assessment, IPP, and prognosis which had a range of successful applications of a step. For “Patient History” and “Patient Examination,” seven steps were applied by more than 95% of students. For IPP (naming another discipline), less than half applied the step for both groups. This step showed the lowest performance of any step. For “Risk Assessment,” the range of student applications of a step ranged from 65% to 100%. For compliance capacity, onset of disease status, and risk for disease progression, 100% of students in both groups applied the step. For social barriers, 92% applied the step for periodontitis risk assessment and 75% applied the step caries risk assessment. For behavior and diet control, 100% of students applied the step for caries, and 81% and 71% applied the step for periodontitis. Related to “Treatment,” with the exception of “Prognosis” for periodontitis with 68.4% of students applying the step, 100% of students applied the three remaining steps for Periodontitis assessment and at least 87.5% of students applied each of the four steps for the caries group. For EBD, 100% applied the step for both groups.

**Table 1:** Learning guide for patient assessment to include risk assessment for dental caries and periodontitis. Performance assessment is based on the application of respective steps. Some steps are more procedural, and some call more for thinking and judgment.

Learning guide (Steps for the thought process)		Performance assessment (*n* = 78)			
		Periodontitis Assessment (*n* = 38)		Caries Assessment (*n* = 40)	
		Applied	Missed	Applied	Missed
**Patient history**	– Medical history – Medications – Dental history – Social history	38 (100%) 37 (97.6%) 38 (100%) 37 (97.6%)	0 1 (2.6%) 0 1 (2.6%)	40 (100%) 40 (100%) 40(100%) 39 (97.5%)	0 0 0 1 (2.5%)
**Patient examination**	– Clinical exam – Radiographic exam – Interdisciplinary needs attended – Chief concern	38 (100%) 38 (100%) 17 (44.7%)37 (97.6%)	0 0 21 (55.3%) 1 (2.6%)	40 (100%) 39 (97.5%) 8 (20%) 40 (100%)	0 1 (2.5%) 32 (80%) 0
**Risk assessment**	– Patient expectations – Compliance capacity – Social barriers – Onset of disease is visible – Risk for disease progression – Behavioral conditions (psychological/drugs) – Diet/nutrition: controlled/uncontrolled	29 (76.3%) 38 (100%) 35 (92.1%) 38 (100%) 38 (100%) 31 (81.6%) 27 (71.1%)	9 (23.7%) 0 3 (7.9%) 0 0 7 (18.4%) 11 (28.9%)	26 (65%) 40 (100%) 30 (75%) 40 (100%) 40 (100%) 40 (100%) 40 (100%)	14 (35%) 0 10 (25%) 0 0 0 0
**Prioritize conditions before treatment**		38 (100%)	0	39 (97.5%)	1 (2.5%)
**Prognosis without treatment**		38 (100%)	0	40 (100%)	0
**Treatment**	Alternatives provided	38 (100%)	0	38 (95%)	2 (5%)
	Rationale for selected treatment	38 (100%)	0	39 (97.5%)	1 (2.5%)
	Duration of therapy	38 (100%)	0	39 (97.5%)	1 (2.5%)
	Prognosis with treatment	26 (68.4%)	12 (31.6%)	35 (87.5%)	5 (12.5%)
**Evidence‐based dentistry**		38 (100%)	0	40 (100%)	0
**Evaluation of outcomes**		38 (100%)	0	37 (92.5%)	3 (7.5%)
**Student self‐assessment/biases**		18 (47.4%)	20 (52.6%)	30 (75%)	10 (25%)

Risk assessment is reported in the context of the overall learning guide. The level of performance for steps not associated with risk assessment were recorded on whether the student applied the step in the learning guide. On the other hand, level of performance for steps in risk assessment were reported at a level of grasping the concept.

### 3.2 Periodontal Risk Assessment

Within the patient population in the Periodontics sessions, students reported patients with smoking (*n* = 13), diabetes (*n* = 4), and/or obesity (*n* = 1). From results posted by students following the template learning guide, it was found that patients with these medical conditions were significantly correlated with lower compliance capacity (*p* = 0.013*), increased disease onset (*p* < 0.001***), disease progression (*p* < 0.001***), and poor prognosis (*p* = 0.001***) (Table 2). Interestingly, the number of erratic/non‐compliant periodontal patients with evident signs of active disease, higher risk for disease progression, and less favorable prognosis were among patients with smoking, diabetes, or obesity as denoted by students.

**Table 2:** Data on periodontitis risk assessment from student reports. Patients without smoking, diabetes, or obesity are more likely to be compliant (*p* = 0.013*), have minimal disease (*p* < 0.001***), be at lower risk (*p* < 0.001***), and have better prognosis (*p* = 0.001***).

Variables	Smoking/Diabetes/Obesity:	Smoking/Diabetes/Obesity:	Wald Chi‐square test	*p‐*value
	Yes, *N* (%)	No, *N* (%)	Chi‐square value	
Compliance				
Yes	7 (30.4)	16 (69.6)	6.70	0.013*
No	11 (73.3)	4 (26.7)		
Disease				
Not Present	5 (21.7)	18 (78.3)	15.35	< 0.001***
Present	13 (86.7)	2 (13.3)		
Risk				
Low	6 (24.0)	19 (76.0)	16.01	< 0.001***
High	12 (92.3)	1 (7.7)		
Prognosis				
Favorable	6 (25.0)	18 (75.0)	13.07	0.001***
Not Favorable	12 (85.7)	2 (14.3)		

*p* > 0.05, not significant.

* *p* ≤ 0.05

*** *p* ≤ 0.001.

### 3.3 Caries Risk Assessment

Caries risk assessment data as posted during PowerPoint presentations (Table 3), students categorized patients by risk level (minimum or high) according to compliance, disease presence, behavior controlled or not, diet controlled or not, and prognosis were all statistically significant from *p* = 0.006** to *p* < 0.001***. Patients who indicated having positive compliance, absence of disease, controlled behavior, and diet had minimum risk and minimum disease progression.

**Table 3:** Data for caries risk from student reports. Patients with minimum risk/disease progression (Level 1) are more likely to be compliant (*p* = 0.001***), to not have disease onset visible (*p* < 0.001***), have controlled behavior condition (*p* = 0.006**), have controlled diet/nutrition (*p* < 0.001***) and have more favorable diagnosis/prognosis (*p* = 0.005**) compared to risk level other than 1.

Variables	Risk Level 1	Risk Level 2 or 3	Wald chi‐square test	*p* value
	*N* (%)	*N* (%)	Chi‐square value	
Compliance				
Compliant	10 (71.4)	4 (28.6)	12.56	0.001***
Not	4 (15.4)	22 (84.6)		
Onset Disease visible				
Not Present	10 (66.7)	5 (33.3)	10.58	0.002**
Present	4 (16.0)	21 (84.0)		
Behavior conditions				
Controlled	13 (56.5)	10 (43.5)	11.02	0.006**
Not	1 (5.9)	16 (94.1)		
Diet Nutrition				
Controlled	13 (72.2)	5 (27.8)	19.93	< 0.001***
Not	1 (4.5)	21 (95.5)		
Diagnosis Prognosis				
Favorable	12 (57.1)	9 (42.9)	9.53	0.005**
Not	2 (10.5)	17 (89.5)		

*p* > 0.05, not significant.

** *p* ≤ 0.01

*** *p* ≤ 0.001.

An anecdotal observation suggested a disconnection between the student's objective assessment of risk factors and a less objective assessment of a patient's prognosis. As such, students tended to give a patient an optimistic prognosis, among higher‐risk patients as “favorable with compliance” but recorded that the patient was less than compliant.

## 4 Discussion

The present project sought to combine risk assessment for caries and periodontitis based on the common denominator of managing the person rather than mitigating the disease consequences through procedures or on treating the disease based on topical medications. Additionally, the tools for successful interventions are limited for high‐risk or noncompliant people. Thus, the practitioner formally or intuitively develops an analysis for dealing with high‐risk people with recurring disease.

To the knowledge of the authors, there is no risk assessment combining features of the model tested in this project in the literature and including convergence of risk assessment thought processes for caries and periodontitis; explicit assessment of patient capacity to subscribe to professional recommendations; addressing the disease at the levels of managing the person, treating the disease, and mitigating consequences of disease; converting the risk assessment thought process into the learning outcome, learning guide and assessment instrument. Results from the present project were promising to continue the development of this emulation model. While caries and periodontitis each as a microbial basis, the risk factors are different, thus an imbalance for some risk factors such as diet; for caries, sweets are the major dietary factor with sweets playing a lesser role for periodontitis.

For the primary purpose of combining risk assessment for caries and periodontitis into a single risk assessment using an emulation model for critical thinking, students applied each step in the learning guide at a high percentage for both diseases. The combination of risk assessments was considered successful with only small percentages of individual omissions when comparing results for risk assessments for caries and periodontitis. The focus on risk assessment as part of overall person/patient analysis was an attempt to engage the student in critical thinking at a pivotal point. With the literature being sparse on critical thinking and its assessment, this project is also a call to action for a greater focus on these subjects.

While exploring factors in student analysis contributing to designation of higher risk and lower risk for caries and periodontitis using this model, pivotal findings may lead to adjustments in learning guide design. A challenge for the educator is the line or gap from performing risk assessments on relatively healthy people to performing risk assessments on compromised people (T. W. Craig et al. 2022; Shabayek et al. 2023). The complexity of risk assessment multiples from a healthy person to a compromised person, whether the person is compromised medically, socioeconomically, or behaviorally (Chavis and Macek 2022; Murray et al. 1996; Salomon et al. 2012) For both diseases, healthy people are aware of how to stay healthy – regular follow‐ups, practice good oral hygiene habits, cariogenic diet restriction, adherence to medication regimens, smoking cessation, etc. Compromised people may lack of compliance whether through inability or unwillingness (T. W. Craig et al. 2022; Shabayek et al. 2023). Some of the perspectives factored in by the master clinician include discernment of disease presence and progression, patient variables, the expertise of other health professionals, prognosis for the patient, alternative treatments, application of the best science for this patient, and assessing one's biases in planning care – a set of thought processes built on critical thinking (T. W. Craig et al. 2022; Johnsen et al. 2012; Marshall et al. 2017). The challenge becomes even greater among a cohort of high‐risk individuals with recurring disease and history of unsuccessful interventions (Chavis and Macek 2022; Murray et al. 1996; Salomon et al. 2012). This is one of the main reasons why a person‐centered approach is important.

Interventions for high‐risk people with recurring disease is still largely unresolved (Shabayek et al. 2023). In a student sample of patients largely divided between high‐risk people and relatively healthy people seeking routine specialty procedures (e.g. dental implants, restorations in the esthetics zone), the authors were surprised to see how sharply the students discerned the higher‐risk people, both for caries (based on diet controlled or not) and periodontitis (based on diabetes, smoking, obesity). While unanticipated, the population sampled gave students experience in differentiating higher from lower‐risk people. We submit that enough thought progress has been made to continue the discussion on risk models for caries and periodontitis, considering the elements consolidated in this project. Specifically, the concept of probing analyses with binary conclusions is worth further study for the educator. Also, the correlation of treatment plans with student assessments on future dental care will be valuable for the student before beginning extensive dental treatment. Dental students will face this scenario on a daily basis in private practice and it is essential as an educator to prepare them for this moment.

Within the limitations of the present study, an emphasis on students' critical thinking abilities in risk assessment and other areas were not reported in detail. Student's procedural capabilities in gathering data were not a focus beyond what is reported in the overall context of the learning guide. Effectiveness of applying EBD to the patient was not reported beyond recording the student applying steps in EBD. Topics raised in this project for potential follow‐up in further studies included the methodology of probing analysis with binary decisions, the association of treatment and health, and the balance within dental education on relative emphasis for the spectrum of mitigating disease consequences, treating the disease, or managing the person.

## 5 Conclusions

A combined learning outcome, learning guide, and assessment instrument is based on an emulation model for critical thinking that merges risk assessment for caries and periodontitis. Such an approach was found to show promise for continued development. The combined assessment evaluated the risk level of the person more than the level of the disease mitigation or treatment of the disease. The project was successful in combining the learning guides for caries and periodontitis and resulted in objective adjustments emphasizing the students' ability to assess higher‐risk individuals. The present model offers enough power to analyze a relatively healthy person as well as a high‐risk person with recurring disease.

## Author Contributions

David Johnsen, Justine Kolker, and Megumi Williamson contributed to the conception, preparation, and design of the study. David Johnsen, Aditi Jain, and Carlos Garaicoa‐Pazmino were faculty examiners during student‐led presentations and responsible for data collection. Wei Shi performed the data analysis. All authors were involved in the manuscript preparation and approved the final version of the manuscript.

## Conflicts of Interest

The authors declare no conflicts of interest.

## Supporting information

cre2.20240239‐File004.

## Data Availability

The data that support the findings of this study are available from the corresponding author upon reasonable request.
