# Supplementary material for: An Emulation Model Combining Caries and Periodontitis Risk Assessment With Student Performance Report: An Exploratory Project
Source: Clin Exp Dent Res. 2025 Apr 30;11(3):e70135. doi: 10.1002/cre2.70135 (PMC12042111; doi:10.1002/cre2.70135)
Supplement: Supplementary file 1 — cre2.20240239‐File004. [file CRE2-11-e70135-s001.docx]

**A = applied the step; G = Grasped the concept of the step or group of steps**

| **PATIENT DATA** | | |
| --- | --- | --- |
| **Steps on the thought process** | **Performance assessment** | **Comments** |
| **Patient history**   1. Medical history (e.g., Diabetes/Smoking, Obesity) 2. Medications 3. Dental history 4. Social history | **G**  **A**  **A**  **A**  **A** |  |
| **Patient examination**   1. Clinical exam 2. Radiographic exam 3. Interdisciplinary needs attended 4. Chief concern 5. Which data are most important? | **G**  **A**  **A**  **A**  **A**  **A** |  |
| **Risk assessment**   1. Patient expectations 2. Compliance capacity 3. Social barriers 4. Onset of disease is visible 5. Risk for disease progression 6. Behavioral conditions (psychological/drugs) 7. Diet/nutrition problems | **G A**  High / Low  Compliant/Erratic/Non-compliant  Transportation/Finances/Support  Present/Non-present  -Minimal risk  -High risk with minimal Disease Progression  -High risk and disease Progression  -ROHD  Controlled/Uncontrolled  Controlled/Uncontrolled |  |
| **Diagnosis –** Prioritize conditions   1. Available data are complete 2. Diagnosis is accurate/complete | **G**  **A**  **A** |  |
| **Etiological factors recognized** | **G A** |  |
| **Prognosis**   1. Overall 2. Prognosis (w/o treatment) | **G A**  Favorable, Questionable, Unfavorable, Hopeless  Onset for Rapid Oral Health Deterioration (ROHD)? |  |
| **Treatment**   1. Alternatives provided 2. Rationale for selected treatment 3. Duration of therapy 4. Prognosis | **G**  **A**  **A**  **A**  **A** |  |
| **Evidence-Based Dentistry**   1. Assess/Ask: PICO question 2. Acquire: Quality/Depth 3. Appraise: Article selection 4. Apply: Relevance to PICO/Patient | **G**  **A**  **A**  **A**  **A** |  |
| **Evaluation of treatment outcomes**   1. Understanding between Expected vs Final outcomes: Compliance | **G A** |  |
| **Presentation style/factors**   1. Duration 2. Difficulty 3. Clarity 4. Preparation | Acceptable/Non-acceptable  Easy/Routine/Difficult/Very Difficult  Comprehensive/Succinct/Visual  Well-prepared/Acceptable/Non-acceptable |  |
| **Student self-assessment/biases**   1. Symptoms/Disease/Person | **G A** |  |
